# Intermittent aspiration of pharyngeal secretion for re-intubation prevention

**DOI:** 10.1186/cc10738

**Published:** 2012-03-20

**Authors:** T Nakamura, O Nishida, J Shibata, N Kuriyama, Y Hara, M Yumoto

**Affiliations:** 1 Fujita Health University School of Medicine, Toyoake, Japan

## Introduction

**Figure 1:**
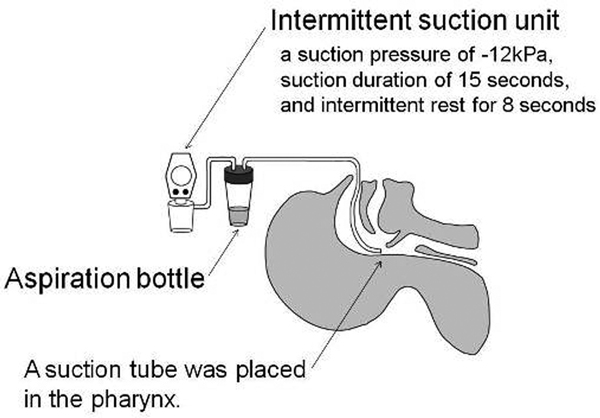
**Intermittent aspiration of pharyngeal secretion**.

The inability of extubated patients to clear oropharyngeal secretion increases the risk of re-intubation. To eliminate excessive oropharyngeal secretion, we devised a suctioning method: intermittent aspiration of pharyngeal secretion (IAPS). IAPS is a simple, low-cost technique utilizing an intermittent suction unit and a common suction tube (Figure 1), which may reduce the risk of re-intubation on extubated patients requiring supraglottic airway management.

## Methods

A retrospective study was performed on 24 patients who received IAPS after extubation from June 2009 to May 2011. A suction tube was placed in the pharynx after extubation. The same suction unit used in intermittent subglottic secretion drainage was applied. IAPS is effective for patients with large amounts of oropharyngeal secretion (A), patients with poor laryngopharyngeal function (B), and patients unable to expel viscous sputum (C). Efficacy of IAPS in each of these patient groups was studied.

## Results

The average age was 64.3 ± 17.8 years, APACHE II score 21.0 ± 7.7, and SOFA score 8.4 ± 3.1. Six patients were diagnosed with A, three with B, two with C, and others had multiple diagnoses. Combinations with NPPV or cricothyroidotomy were also successful. Of the patients who required re-intubation, four were re-intubated for reasons other than aspiration. Two had possibly aspirated. Among patients receiving IAPS, the rate of re-intubation due to oropharyngeal aspiration was 8.3%. No major complication was observed.

## Conclusion

IAPS is a potential method for supraglottic airway management after extubation that may reduce the re-intubation risk. IAPS is a simple method requiring common instruments. Combined effects of IAPS with NPPV or cricothyroidotomy can modify airway management. IAPS is a temporary method in which the exact timing for re-intubation should not be missed. To successfully apply IAPS and reduce aspiration, the suctioning method, duration of application and position of the suctioning tube should be further optimized.

